# Supplementary material for: Ag Catalysts Supported on CeO2, MnO2 and CeMnOx Mixed Oxides for Selective Catalytic Reduction of NO by C3H6
Source: Nanomaterials (Basel). 2023 Feb 26;13(5):873. doi: 10.3390/nano13050873 (PMC10005331; doi:10.3390/nano13050873)
Supplement: Supplementary file 1 [file nanomaterials-13-00873-s001.zip › nanomaterials-2225807-supplementary.pdf]

nanomaterials

Supplementary Materials

Ag Catalysts Supported on CeO_2, MnO_2 and $CeMnO_x$ Mixed Oxides for Selective Catalytic Reduction of NO by C_3H_6

Eleonora La Greca [1], Tamara S. Kharlamova [2], Maria V. Grabchenko [2], Luca Consentino [1], Daria Yu. Savenko [2], Giuseppe Pantaleo [1], Lidiya S. Kibis [3], Olga A. Stonkus [3], Olga V. Vodyankina [2,*] and Leonarda Francesca Liotta [1,*]

[1] Institute for the Study of Nanostructured Materials (ISMN), (Italian) National Research Council (CNR), Via Ugo La Malfa 153, 90146 Palermo, Italy
[2] Laboratory of Catalytic Research, Tomsk State University, Lenin Ave. 36, 634050 Tomsk, Russia
[3] Boreskov Institute of Catalysis SB RAS, Lavrentiev Ave. 5, 630090 Novosibirsk, Russia

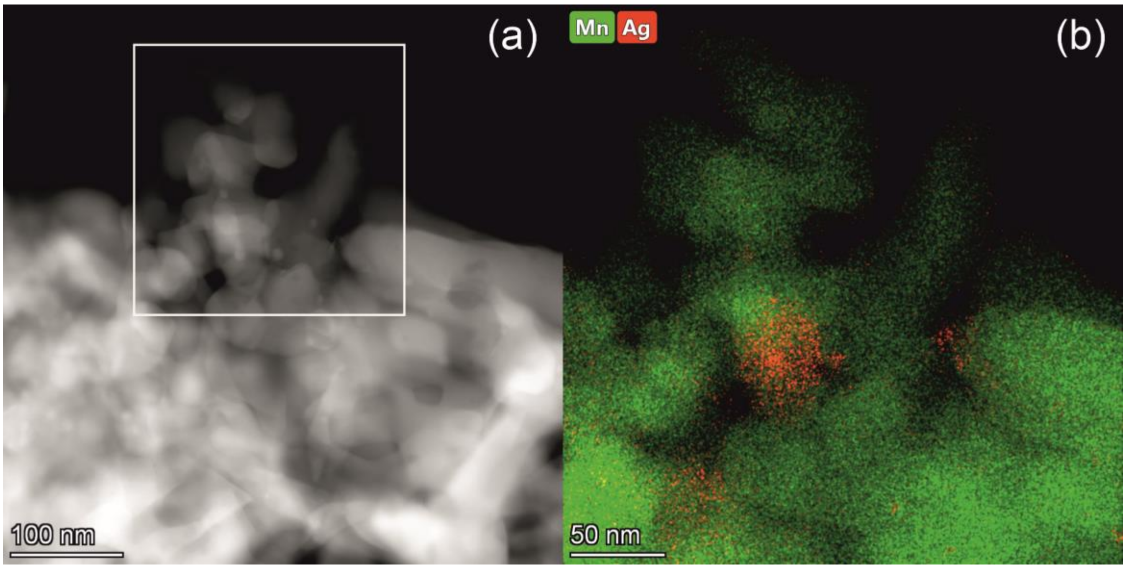

Figure S1. HAADF STEM **(a)** and high resolution EDX mapping of indicated region **(b)** for the Ag/MnO_x sample.

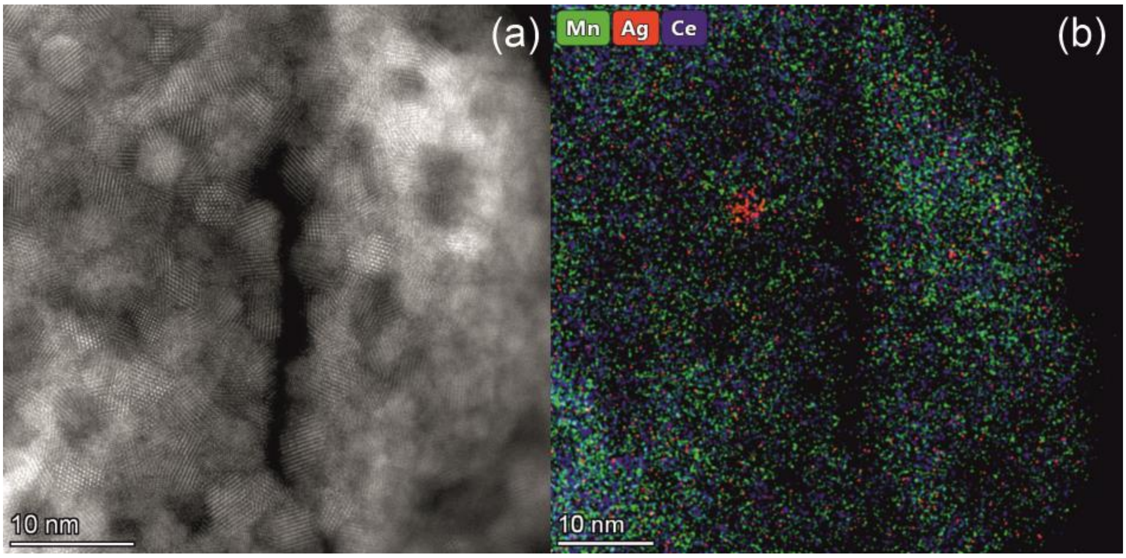

Figure S2. HRTEM image **(a)** and high resolution EDX mapping **(b)** for the Ag/$CeMnO_x$ sample.

Results of Studies of Aged Samples

The results of the study of aged samples by the low-temperature adsorption method are presented in Table S1. The presented data indicate that under aging conditions, there is a strong change in the textural characteristics of the catalysts as a result of sintering. For Ag/CeO_2 and Ag/$CeMnO_x$ samples, a decrease in the SSA by a factor of 2 is observed, while the SSA decrease by a factor of 2 is observed for the Ag/MnO_x sample.

Table S1. Specific surface area (SSA) and total pore volume (V) of supports and catalysts determined by low-temperature nitrogen adsorption/desorption data.

**Sample**	**SSA., m²/g**	**V, cm³/g**
Ag/CeO_2	34	0.13
Ag/CeO_2 (aged)	14	0.12
Ag/MnO_x	12	0.12
Ag/MnO_x (aged)	3	0.01
Ag/$CeMnO_x$	47	0.22
Ag/$CeMnO_x$ (aged)	21	0.16

Table S2 shows the phase composition of the samples and the characteristics of their crystalline phases according to XRD data. According to the XRD data, in the aged Ag/CeO_2 sample, only a phase with a fluorite structure is present with a crystal lattice parameter, a = 5.41 Å. At the same time, a noticeable increase in the size of crystallites of the fluorite phase up to 43 nm is observed for the aged Ag/CeO_2 sample in comparison with the initial catalysts.

For the aged Ag/MnO_x sample, the formation of one cubic phase of manganese oxide Mn_2O_3 with a crystal cell parameter of 9.40 Å and a crystallite size of ~97 nm is observed.

In the aged Ag/$CeMnO_x$ sample, according to the XRD data, there are two crystalline phases: a relatively finely dispersed CeO_2 phase with a fluorite structure and a rough phase of manganese oxide Mn_2O_3.

Table S2. Phase composition of aged samples and characteristics of their crystalline phases according to XRD data.

**Sample**	Phase composition		Structural parameters		D_{XRD}, nm
	Phase	Content, wt.%	S.G.	*a*, Å	
**Ag/CeO_2 (aged)**	fluorite	100	Fm-3m	5.41	43
Ag/MnO_x (aged)	Mn_2O_3	100	Ia-3	9.40	97
Ag/$CeMnO_x$ (aged)	fluorite	72	Fm-3m	5.40	13
	Mn_2O_3	28	Ia-3	9.40	n.a.*

*The correct determination of a full width at half maximum is impossible.
